# Characterization of Phenolic Compounds and Their Contribution to Sensory Properties of Olive Oil

**DOI:** 10.3390/molecules24112041

**Published:** 2019-05-28

**Authors:** Vasilisa Pedan, Martin Popp, Sascha Rohn, Matthias Nyfeler, Annette Bongartz

**Affiliations:** 1 Life Sciences and Facility Management, Zurich University of Applied Sciences, 8820 Wädenswil, Switzerland; martin.popp@zhaw.ch (M.P.); matthias.nyfeler@zhaw.ch (M.N.); annette.bongartz@zhaw.ch (A.B.); 2 Institute of Food Chemistry, Hamburg School of Food Science, University of Hamburg, Grindelallee 117, 20146 Hamburg, Germany; rohn@chemie.uni-hamburg.de

**Keywords:** olive oil, LC-MS analysis, phenolic compounds, polyphenols, sensory profiling, aroma description

## Abstract

Olive oil is not only known for its pungent, bitter, and fruity taste, but also for its health potential, which is often hypothesized to depend on its phenolic compounds. One hundred extra virgin olive oil samples (monocultivaric as well as blends of varieties) were assessed with regard to their sensory properties and phenolic compound composition. Nineteen phenolic compounds have been determined and correlated with sensory data. In all olive oil samples, oleocanthal and oleacein were the most abundant phenolic compounds, with average amounts of 77.9 mg/kg and 41.8 mg/kg, respectively. The highest correlation coefficient between a sensory descriptor and the phenolic compounds was found for the bitter taste sensation and the total phenolic content with r = 0.72 and in particular, for 3,4-DHPEA-EA, with r = 0.57. Intensity plots were assessed for the three main sensory descriptors fruitiness, bitterness, pungency, and for the quality factor harmony, which is associated with the degree of ripeness aroma of olive oil. Positive correlations for the aroma descriptors freshly cut grass, leaves, and nuts, and the phenolic compounds were especially observed for oleoside 11-methylester and vanillic acid. The present study provides a comprehensive database of phenolic compounds in olive oils from six different varieties and seven countries.

## 1. Introduction

The global production of olive oil has increased from 1.4 million tons to 3.3 million tons over the last three decades [1]. Not only because of its fruity and aromatic notes, higher consumption levels and popularity (especially in Northern Europe) can also be attributed to the advertised health-promoting effect of olive oil, which has been well described by diverse studies of different disciplines [2,3,4].

Olive oil consists of over 98% triacylglycerols, with oleic acid as the dominating esterified fatty acid. Minor components such as free fatty acids, mono- and diacylglycerols, a wide range of lipids such as hydrocarbons, sterols, aliphatic alcohols, tocopherols, pigments, and phenolic compounds represent about 2% of the oil [5]. Some of these compounds contribute to the unique sensory characteristics of olive oil. The phenolic compounds are of particular interest. They are not only responsible for the antioxidant activity [6], stabilizing the products endogenously for an enhanced shelf-life [7], but they provide also sensory properties such as bitterness and pungency [8].

Olive oil in general displays a broad spectrum of sensory descriptors with the main attributes being fruitiness, bitterness, and pungency. For a comprehensive specification, single flavor descriptors such as freshly cut grass, tomato, artichoke, leaves, nuts, apple, banana, tropical fruits, and herbs that are part of the main attribute fruitiness, should be also taken into account. The presence of these aroma compounds in olive oil depends, among other factors such as origin, variety, cultivation, and processing, to a great extent on the degree of ripeness (harvest time) of the raw material. Depending on the olive maturity, manufactured olive oils can be grouped as green, green/ripe, or fully ripe. Furthermore, the overall intensity of the fruitiness of olive oils can be classified as light, medium, and intense. The presence and interaction of all descriptors and their corresponding sensory perceptions contribute to the complexity and balance of olive oils, which is defined as harmony.

Ecophysiological parameters, in particular, have a significant impact on the total phenolic content (TPC) of olive oil. Being typical for a natural product, the TPC differs with region, variety, growing conditions (soil, plant nutrition etc.), maturation, harvest time, and processing. These factors, among others, lead to a wide TPC range of 50–1000 mg/kg, whereby the average content is between 100–300 mg/kg [9]. Phenolic compounds in olive oil are reported to cause a bitter, astringent, and pungent sensation [10]. A study carried out by Gawel and Rogers [11] classified olive oils by means of their TPC, with olive oils providing a content of less than 80 mg/kg being described as ‘mild’, and over 440 mg/kg as ‘robust’. Sensory evaluations performed by Boskou and co-workers [12] reported a dominating bitter taste sensation for olive oils with a TPC content higher than 300 mg/kg.

The TPC consists of various phenolic classes, such as phenolic acids (vanillic, coumaric, caffeic, protocatechuic, *p*-hydroxybenzoic, ferulic acid), lignans (acetoxypinoresinol, pinoresinol), flavones (apigenin, luteolin), flavone glycosides (luteolin-7-*O*-glucoside, apigenin-7-*O*-glucoside), phenolic alcohols (tyrosol, hydroxytyrosol), and secoiridoids (oleacein, oleocanthal, oleuropein, *p*-HPEA-EA) [13]. The predominant phenolic compound found in olive oil is oleuropein and its hydrolytic breakdown products, hydroxytyrosol, and tyrosol [14]. Phenolic acids are reported being in a concentration range of 0.01–1.7 mg/kg. Still, the single contribution of each individual phenolic compound to the total bitterness is not fully evaluated. However, some of these individual phenolic compounds exhibit specific sensory properties. Studies performed by Gutierrez-Rosales and co-workers [15] indicated that oleacein and the dialdehydic form of decarboxymethylligstroside aglycone contribute to the bitter taste. Additionally, Andrewes and co-workers [16] identified that the dialdehydic forms of deacetoxyoleuropein and diacetoxyligstroside aglycone, isomers of ligstroside, and oleuropein aglycons provide bitter and astringent sensations.

A systematic study on a large number of samples and phenolic compounds and the sensory properties has not yet been reported in the literature. Consequently, the present study aimed at a comprehensive analysis of olive oils and their sensory properties as well as the determination of phenolic compounds contents, profiles, and their correlation with the sensory properties. Within this study, a selection of olive oils (*n* = 100) were analyzed with regard to their origin and genotype. Subsequently, the sensory descriptors fruitiness, bitterness, pungency, harmony, and flavor descriptors were linked to the concentration of the phenolic compounds. Possible correlations and interactions were derived from applying different statistical analytical approaches.

## 2. Results and Discussion

### 2.1. Total Phenolic Content

Olive oil polyphenols contribute to the protection of low-density lipoproteins from oxidative damage. For this reason, the European Food Safety Authority has attributed a health claim, confirming that a serving of 20 g olive oil should contain at least 5 mg hydroxytyrosol and its derivatives (e.g., oleuropein and tyrosol). Extra virgin olive oil (EVOO), with a ‘natural polyphenol level’ above 250 mg/kg and therefore comply the EU Health Claim can indicate this by calling their olive oil ‘Healthy Extra Virgin Olive Oil’ [17]. Logically, the method of choice to determine the total phenolic content can be a photometrical method like Folin-Ciocalteu assay. It must be noted, that there is no consent on the determination procedure, whereby Folin-Ciocalteu has been proven as a stable marker for VOO stability and taste characteristics.

In the present study, the amount of TPC in EVOO varied according to the sample and ranged from about 52–315 mg GAE/kg (Table 1). Only four out of 100 samples had a TPC above 250 mg GAE/kg. These results are in accordance with previous research on olive oil done by Caporaso et al. [18]. There, just three out of 32 EVOO could be provide with a valid health claim. Other studies have identified similar contents with olive oils ranging from 40–530 mg GAE/kg oil [6].

The interest in phenolic compounds is because of their certain contribution to olive oil bitterness and pungency [19]. Servili et al. [19] postulated for Italian olive oil a taste perception to their TPC as low with 50–200 mg GAE/kg, medium with 200–500 mg GAE/kg and high with 500–1000 mg GAE/kg (gallic acid equivalent).

According to Angerosa et al. [20], TPC is related to bitterness, whereby a TPC < 220 mg CAE/kg (caffeic acid equivalent) in olive oil is perceived as non-bitter, 220 to 340 mg CAE/kg as lightly bitter, with 340 to 410 mg CAE/kg as bitter, and >410 mg CAE /kg as very bitter [21]. TPC in virgin olive oil expressed as gallic acid equivalent range in value from 50–800 mg/kg. Most oils have TPC around 180 mg/kg. The cultivar, the system of extraction, and the conditions of processing are critical factors for the TPC [5].

Other studies have already investigated relationships between sensory properties and individual chemical compounds [22]. However, the role of each individual phenolic compound to the total bitterness is not yet clear. Furthermore, much more interesting is the recognition of a high phenolic olive oil not only due to TPC and bitter, pungent taste but also to its possible correlation of sensory attributes and individual phenolic compounds. Therefore, the present study worked on a predictive model to understand the influence of the TPC and individual phenolic compounds with the sensory properties of olive oils, like fruitiness, bitterness, pungency, and the quality factor harmony.

Therefore, not surprisingly, the present study has evaluated a correlation coefficient of TPC and bitterness with r = 0.69, and a correlation coefficient of TPC and pungency with r = 0.54 (Supplementary Material).

In general, most of the sensory studies used individual phenolic compounds isolated from olive oil by using liquid-liquid extraction with further separation using RP-HPLC followed by fraction collection. Hereby, expert panelists determined relationships between oleocanthal and oleacein and the burning/pungent note in the olive oil [16].

The present study could identify *p*-HPEA-EA and oleuropein as phenolic compounds contributing most to the bitter and pungent sensation, with *p*-HPEA-EA and correlation coefficient of 0.46 for bitter and 0.37 for pungent, and oleuropein with a correlation coefficient of 0.57 for bitter and 0.40 for pungent, respectively (Supplementary Material).

Bioactive properties have been hypothesized for hydroxytyrosol in different in-vivo and in-vitro studies with health-promoting abilities targeting inflammation, LDL oxidation, oxidative stress, cancer etc. [23]. The present study showed that only three out of hundred olive oils provide a content of higher than 1.5 mg hydroxytyrosol and its derivatives (e.g., oleuropein and tyrosol) per serving, which still does not meet the requirements of the official European health claim with 5 mg hydroxytyrosol and its derivatives per serving.

However, comparatively polar phenolic compounds are responsible for the sensory characteristics, whereby the totality of all biochemical compounds plays an important role for the bitter, pungent, and astringent sensation.

#### 2.1.1. Olive Oil Varities

**Figure 1:**
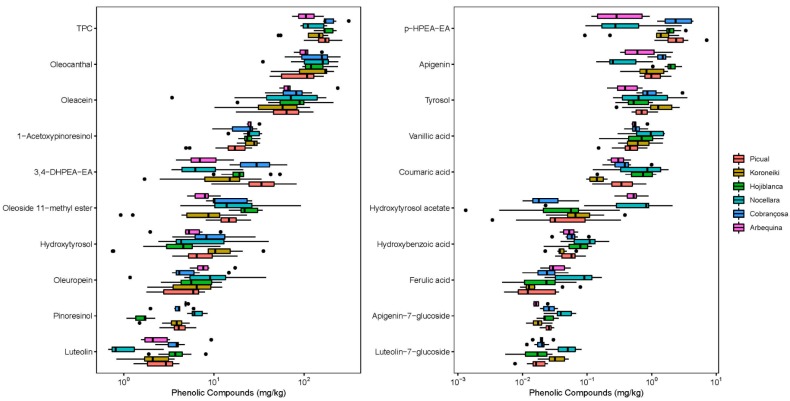
Individual phenolic compounds of oil from six olive varieties.

The variation of phenolic compounds in olive oil influences the taste and nutritional properties. The most direct impact on the phenolic compounds is the choice of the olive tree and therefore olive variety. All of the analyzed phenolic compounds demonstrated distinct differences for the mono-variety olive oils such as Picual (*n* = 17), Koroneiki (*n* = 12), and Hojiblanca (*n* = 10), being the three varieties with the highest number of samples in this study (Figure 1). ANOVA analysis of the TPC of these varieties showed significant differences with *p* < 0.001. From the other varieties there were less than ten samples from Nocellara (*n* = 9), Cobrançosa (*n* = 8), and Arbequina (*n* = 6).

The HSD post-hoc test showed that the cultivar Cobrançosa (mean TPC 202.0 mg/kg) had a significantly higher TPC than Koroneiki (mean TPC 132.4 mg/kg, *p* = 0.01), Nocellara (mean TPC 127.4 mg/kg, *p* = 0.01), or Arbequina (mean TPC 111.7 mg/kg, *p* < 0.01). Furthermore, Hojiblanca (mean TPC 184.6 mg/kg) contained significantly more phenolic compounds than Arbequina (*p* = 0.02); Picual (mean TPC 174.7 mg/kg) significantly more than Arbequina (*p* = 0.04).

The study done by Talhaoui et al. [24] determined the highest total phenolic content of the fresh olive fruit for the Koroneiki cultivar (mean TPC 19,500 mg/kg), followed by Picual (mean TPC 18,500 mg/kg), and at least for Arbequina (mean TPC 3800 mg/kg).

Lignans like pinoresinol and 1-acetoxypinoresinol, allowed a differentiation between the cultivars and were proposed for olive oil authentication, as presented by Brenes and co-workers [25]. Hereby, they found a pinoresinol and 1-acetoxypinoresinol content of 22.9 and 50.1 mg/kg for Hojiblanca, 37.1 and 65.6 mg/kg for Arbequina, and 43.0 and 1.9 mg/kg for Picual virgin olive oils. However, the present study only detected a pinoresinol and 1-acetoxypinoresinol content of 1.7 and 24.4 mg/kg for Hojiblanca, 4.1 and 20.9 mg/kg for Cobrançosa, 4.2 and 16.9 mg/kg for Picual, 3.7 and 25.5 mg/kg for Koroneiki, 6.6 and 26.2 mg/kg for Nocellara, and 4.9 and 26.1 mg/kg for Arbequina olive oils.

#### 2.1.2. Olive Oil Ripening Flavor

As mentioned before, the chemical composition and the quality of EVOO is to a large extent influenced by ecophysiological factors and especially, harvest time. Metabolic processes take place throughout maturation and ripening, which involves changes in triacylglycerols, aliphatic alcohols, and volatile compounds [26]. The phenolic profile changes with maturation of the olives. As shown for blueberries (*Vaccinium corymbosum* L.), a transformation within the different classes of phenolic compounds and significantly accompanies sensory attributes such as color etc. during the development of unripe fruits to fully maturated ones. Thereby, it is often not clear, which time point might be appropriate for an optimum of health beneficial phenolic compounds [27].

**Figure 2:**
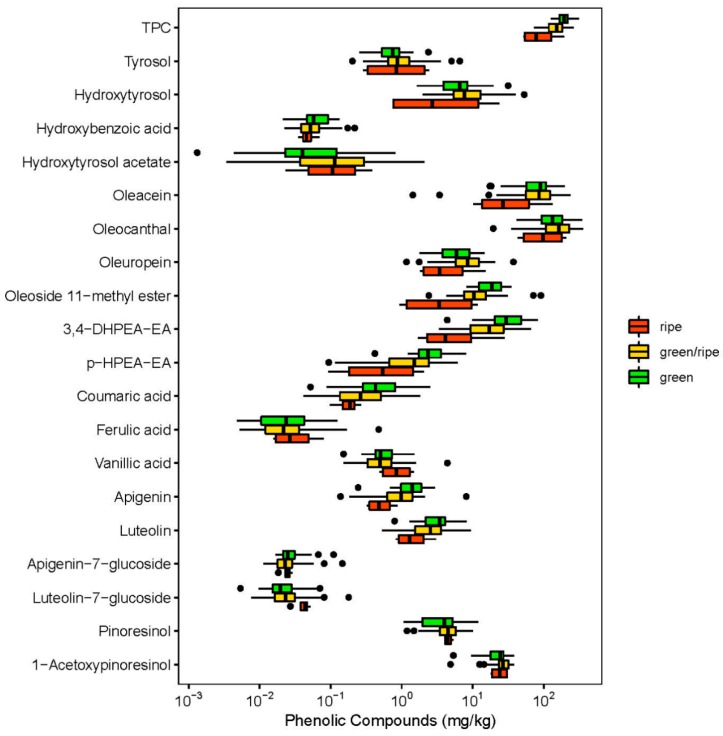
Distribution of individual phenolic compounds between the three different measured ripening flavors ripe, green/ripe and green.

Such variations are further reflected in the taste and flavor characteristics of fruits and vegetables. The present study identified differences in the ripening stages of the processed olive oils and their TPC (Figure 2). The dataset consisted of green (*n* = 30), green/ripe (*n* = 64), and fully ripe (*n* = 6) olive oils. Olive oils with a green flavor impression provided the highest TPC with 194.3 mg/kg; those with a green/ripe aroma a TPC of 157.2 mg/kg and those with a ripe flavor, a TPC of 121.6 mg/kg. These results were not unexpected, as the sensory impression of an unripe olive oil is related to a greenish flavor. A previous study provided information on the ripening stage of the varieties Cobrançosa and Picual: for green olives a TPC of 50.1 mg/kg and 43.5 mg/kg respectively, for semi ripe olives a TPC of 35.3 mg/kg and 28.8 mg/kg, respectively, and for ripe olives a TPC of 34.6 mg/kg, and 31.4 mg/kg, respectively [28]. Thus, impressions such as green, green/ripe, and ripe cannot be directly linked to the ripening stage of olives on the tree, because these impressions are dependent on further factors such as blends of different olive varieties or blends of olives harvested at different times (or the different maturation stages on a single tree). Nevertheless, the present study demonstrated a cascading effect of the TPC and the ripening stage of the olives within the order: green > green/ripe > ripe.

EVOO contains different classes of chemical compounds, whereby most of them are hydrophilic phenol compunds such as phenolic alcohols and acids, flavonoids, lignans, and secoiridoids [13]. One of the first phenolic groups identified in olive oil included phenolic acids such as caffeic, vanillic, syringic, *p*-coumaric, *o*-coumaric, protocatechuic, sinapic, *p*-hydroxybenzoic, and gallic acid. In the present study, a low amount of phenolic acids was identified (Table 1).

A similar tendency as for the TPC could be detected for the individual phenolic compounds, especially hydroxybenzoic acid, oleoside 11-methyl ester, oleuropein, *p*-HPEA-EA, coumaric acid, luteolin, and apigenin. An ANOVA test of the TPC showed significant (*p* < 0.001) differences between the olive oils and different ripening stages. Olive oils described as green had higher TPC than green/ripe oils. Hereby, the TPC showed a significant difference between green and green/ripe (*p* < 0.01) and between green and ripe (*p* < 0.01). In addition, many of the individual phenolic compounds showed a tendency for a correlation with ripening stage: Generally, a lower content was associated with a proceeding ripening stage (Figure 2). The following individual phenolic compounds were also identified as indicators for the ripening stage of the olive: hydroxybenzoic acid, oleoside-11-methyl ester, oleuropein, *p*-HPEA-EA, coumaric acid, luteolin, and apigenin.

Flavonol glycosides such as luteolin-7-*O*-glucoside and apigenin-7-*O*-glucoside and their aglycons luteolin and apigenin are the most abundant in EVOO. As shown in Figure 2, luteolin-7-*O*-glucoside exhibited a slightly increasing trend during the ripening stage of the olive, with a low content of 0.024 mg/kg at the green stage, a medium content of 0.028 mg/kg at the green/ripe stage, and a content of 0.027 mg/kg at late maturity stage. This was in contrast to the aglycon luteolin, which had a high content of 3.24 mg/kg at the green stage, a medium content of 2.89 mg/kg at the green/ripe stage and a low content of 1.65 mg/kg at the ripe stage, illustrating a significant transformation during ontogenic development. Luteolin may originate either from rutin or luteolin-7-*O*-glucoside, whereby a cleavage reaction results in the aglycon form of the flavonol glycoside [29]. The same observations were made for apigenin and apigenin-7-*O*-glucoside.

Artajo and co-workers [30] noted for 1-acetoxypinoresinol and pinoresinol a degradation during the ripening stage. These lignans could be detected in olive fruit but not in olive oil. They proposed a release from the olive fruit during the crushing and malaxation into olive oil due to their lipophilic character.

Wani et al. [23] stated that the phenolic profile of olive oils changes with regard to the maturity stage of the olives. As an example, oleuropein as a predominant secoiridoid in the early stages of olive maturation degrades to hydroxytyrosol. The present study could confirm only rudimentarily this hypothesis. Indeed, oleuropein decreases from green to ripe ripening flavor. However, hydroxytyrosol did not increase during maturation. Not surprisingly, the present study only showed a ripening flavor of the olive oils as an impression of the sensory aroma and not as an agricultural or botanical maturation of the olive oil fruit.

However, the present study, could not confirm these findings with regard to the lignans. Here, the classification into green, green/ripe, and ripe was based on flavor impression and not on typical ripening stages. Nevertheless, a classification in terms of ripening flavor using sensory descriptors can be implemented for distributors or consumers to understand olive oil characteristics and the distribution of individual phenolic compounds in the matrix.

#### 2.1.3. Fruitiness Intensity

**Figure 3:**
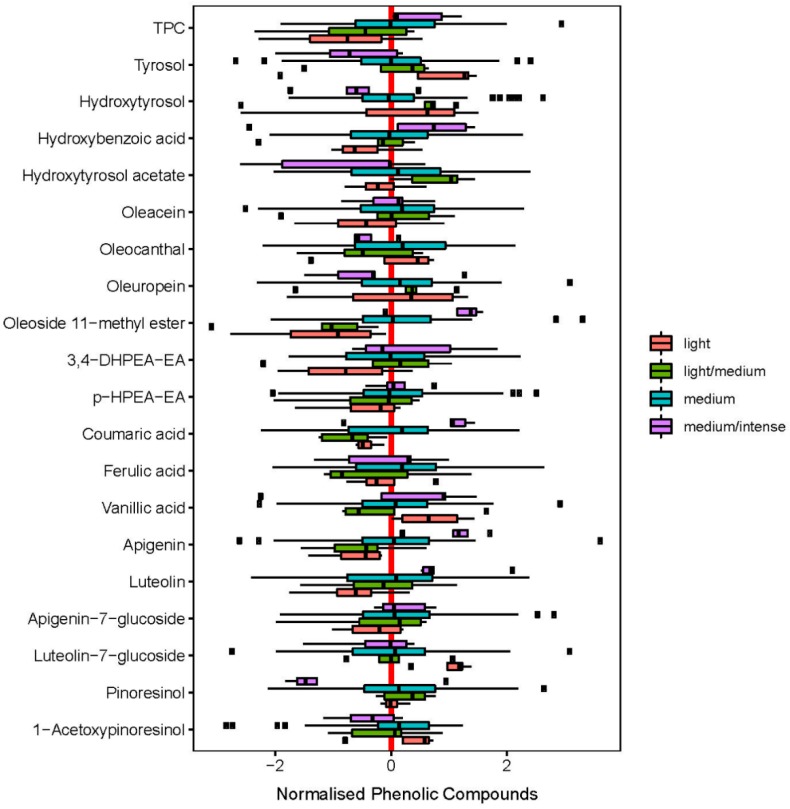
Distribution of individual phenolic compounds between the four different sensory intensity stages light, light/medium, medium and medium/intense in an ascending order.

A further categorization of EVOO can be made with regard to fruitiness intensity. The present study allowed to allocate the samples with regard to their intensity in olive oils that show a light (*n* = 4), light/medium (*n* = 9), medium (*n* = 80), and medium/intense (*n* = 7) fruitiness. Figure 3 shows the different concentrations of TPC in relation to the fruitiness intensity.

Thus, a cascading effect was observed for the intensity of the fruitiness and the TPC of the olive oil with medium/intense > medium > light/medium > light. TPC was able to distinguish between the intensity of olive oils. While medium/intense olive oils had a TPC of 195.7 mg/kg, it was 167.4 mg/kg for medium, 148.8 mg/kg for light/medium, and 126.5 mg/kg for light olive oils.

Likewise, to the TPC, the both flavone aglycones apigenin and luteolin showed a similar cascading behavior within the fruitiness intensity scale. Apigenin and luteolin show also a ‘comparatively good’ correlation with the fruitiness intensity with r = 0.26 and r = 0.20, respectively (Supplementary Material). However, their contribution to their overall impression is rather low due to its content.

Oleoside 11-methyl ester is one of the compounds present in higher concentration in olive oil matrix (Table 1). During the alkali treatment of the olive fruits, oleuropein can be hydrolyzed into hydroxytyrosol, oleoside 11-methyl ester and oleoside, which makes the olive oil less bitter and more delicious [31]. A comparison of the content of oleoside 11-methyl ester showed a significantly higher content (*p* < 0.01) in the medium/intense fruity olive oils, with 27.2 mg/kg (medium/intense) compared to 15.6 mg/kg (medium, *p* = 0.03) and 8.7 mg/kg (light/medium, *p* = 0.04).

The most abundant hydrophilic phenolic compounds are the alcohols hydroxytyrosol and tyrosol. Both contribute to the flavor, stability and nutrition value of EVOO. In the present study, tyrosol was detected with 0.63 mg/kg for the medium/intense fruity olive oil, in contrast to 1.09 mg/kg (medium), 1.51 mg/kg (light/medium) and 1.70 mg/kg (light).

However, especially the both lignans pinoresinol and 1-acetoxypinoresinol are already known as differentiators for specific varieties [32]. Some Picual and Cornicabra fruits showed high levels of both lignans, whereby Arbequina and Empeltre fruits had no detectable 1-acetoxypinoresinol and moderate levels of pinoresinol. Both lignans are principally found in the wooden part of the seed and are released in EVOO during the extraction process.

In the present study, 1-acetoxypinoresinol was analyzed with a level of 30.5 mg/kg (medium/intense) in contrary to 25.8 mg/kg (medium), 20.4 mg/kg (light/medium), and 28.1 mg/kg (light).

#### 2.1.4. Sensory Characteristics

Phenolic compounds have been shown to play an important role in flavor release during the consumption of EVOO. In the present study, the following sensory characteristics were described: leaves (*n* = 94), nuts (*n* = 85), freshly cut grass (*n* = 73), tomato (*n* = 56), apple (*n* = 48), herbs (*n =* 39), banana (*n* = 31), artichoke (*n* = 27), nutshell (*n* = 25), dried nut kernel (*n* = 12), vegetables (*n* = 12), and tropical fruits (*n* = 10). Less frequent, but still notable, were sensory descriptors with the impression of berries (*n* = 4), black tea (*n* = 3), spices (*n* = 2), honey (*n* = 2), cooked vegetables (*n* = 2), malt (*n* = 1), citrus (*n* = 1), and ripe apple (*n* = 1).

**Figure 4:**
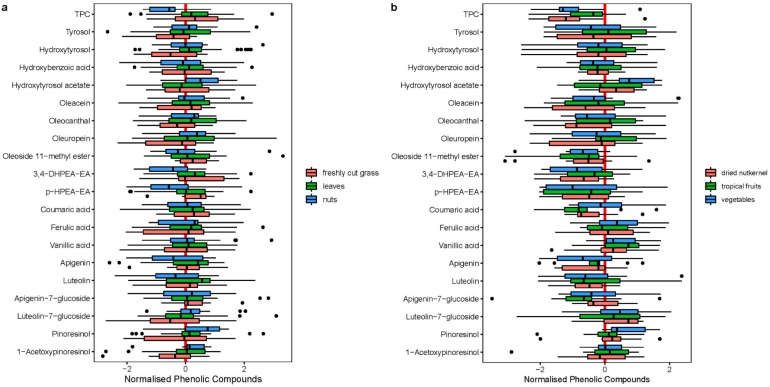
(**a**) Distribution of individual phenolic compounds between the three main occurring sensory descriptors leaves (*n* = 38), nuts (*n* = 22), freshly cut grass (*n* = 13). (**b**) Distribution of individual phenolic compounds between the three rare occurring sensory descriptors dried nut kernel (*n* = 12), vegetables (*n* = 12), and tropical fruits (*n* = 10).

The boxplots of the 20 measured phenolic compounds for the most frequent aroma descriptors: freshly cut grass, leaves, nuts, dried nut kernel, tropical fruits, and vegetables are shown in Figure 4. The TPC and the nineteen measured phenolic compounds for the three most occurring dominant descriptors leaves (*n* = 38), nuts (*n* = 22), freshly cut grass (*n* = 13) are shown in Figure 4a. Olive oils that were described with sensory descriptors like dried nut kernel, vegetables, and tropical fruit had a significantly lower TPC value than the average olive oil (Figure 4b), as shown by a t-test (dried nut kernel *p* < 0.01, vegetables *p* < 0.01, tropical fruit *p* = 0.02). The HSD post-hoc-test showed a significant difference between nutshell (with a mean TPC 200.8 mg/kg) on the one hand and dried nut kernel (mean TPC 127.2 mg/kg) and banana (mean TPC 95.6 mg/kg) on the other.

Oleuropein and its precursor molecule oleoside 11-methylester are known for ten years now. Since then, these compounds have attracted much interest due to their presence during the ripening stage and can be used as indicator for the maturation degree of the fruit. The present study showed that olive oils with low levels of oleoside 11-methylester were associated with aroma descriptors dried nut kernel (10.1 mg/kg), tropical fruits (8.66 mg/kg), and vegetables (7.97 mg/kg). However, olive oils with high levels of oleoside 11-methylester were associated with nuts (16.4 mg/kg) and demonstrate attributes of freshly cut grass (17.1 mg/kg) or leaves (16.2 mg/kg).

Less obvious, but still distinguishable by the sensory descriptors was vanillic acid (Figure 4). The following differentiation was made: freshly cut grass, leaves, nuts with about 0.61 mg/kg, tropical fruit with 0.81 mg/kg and vegetables with 0.76 mg/kg of vanillic acid in olive oil.

### 2.2. Quality Factors Harmony, Fruitiness, Bitterness, and Pungency

From a consumer’s perspective, the differentiation between low, mid and high price EVOOs is not always comprehensible. Diverse suppliers flood the market with olive oils, most of them declared as EVOO, but sometimes with big differences concerning their sensory quality. The frequent use of terms like ‘quality’, ‘mild intensity’, and ‘tasteful’ for marketing purposes impedes the selection of a product by consumers. In this context, the harmony value can be used as an objective sensory descriptor, which works as quality marker [33]. According to the cited study, the category of EVOO should be limited to a harmony value >3.5, whereby olive oils with a harmony value <3.5 should be categorized as virgin olive oil. However, the present study only evaluated olive oils with a harmony level of between 6 and 8.5.

**Figure 5:**
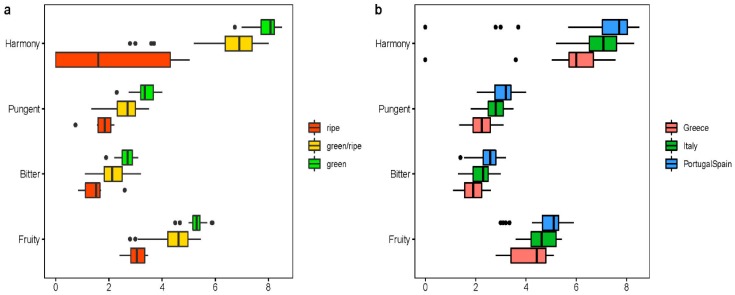
(**a**) The relation of the four main sensory descriptors harmony, pungent, bitter and fruity with the different ripening stages of the olive. (**b**) The relation of the four main sensory descriptors harmony, pungent, bitter and fruity with the different manufacturing countries. Different scales units must be taken into account for the x-axis with fruity, bitter, pungent: 0 = not detectable to 10 = intense; harmony: 0 = no harmony to 5 = standard harmony to 10 = excellent harmony.

The harmony value was found to be increased in relation to the degree of ripeness from green > green/ripe > ripe. Consequently, the highest harmony value was detected within the group of EVOOs with a clearly greenish impression (Figure 5a).

A closer look at the stage of ripening of the olives and oils’ flavor impression revealed that low values of fruitiness, bitterness, and pungency also correlate with the impression of greenish olive oil (Figure 5a). EVOO with a ripe impression are correlated to low values of fruitiness, bitterness, pungency, and harmony. According to Bongartz and Oberg [33], the description green is set to positive olfactory retronasal sensations like freshly cut grass, green leaves, unripe nut, and vegetables like green tomato and artichoke. Ripe olive oil can reminiscent of ripe tomato, cooked artichoke or ripe apple/banana.

Furthermore, a strong Spearman correlation was found between the ripening stage of the olive oils and the sensory descriptors harmony value (r = −0.72), pungency (r = −0.69), bitterness (r = −0.58), fruitiness (r = −0.71) In addition, the Bi-plot (Figure 5) shows that there is a weak clustering of the olive oils by country. Herein, the two Chinese olive oils are outliers whereas the two American ones are rather average compared the other olive oils in the dataset. In Figure 5b there can see a strong tendency, that the Iberian (Portugal and Spain) olive oils have higher values for the sensory parameters fruity, bitter, pungent, and harmony than the Italian ones and these have the same tendency towards the Greek olive oils. Testing with the Kruskal-Wallis-test (*p* = 0.0003827 for fruity, *p* = 3.7 × 10^−5^ for bitter, *p* = 5.2 × 10^−7^ for pungent, *p* = 1.2 × 10^−5^ for harmony) and the Post-Hoc pairwise Wilcoxon-Test shows that all differences and pairwise comparisons are significant. We only compared these three regions as only there are sufficiently many olive oils to be compared with Spain (*n* = 46), Italy (*n* = 29), and Greece (*n* = 18).

However, despite the significance of the sensory descriptors to the country of origin, this result should be considerate carefully. Variation in a country can be as large as between two countries and two varieties. Nevertheless, the present study measured samples of the three countries representing a sufficient number of different mono-cultivars, e.g., eight mono-varieties came from Italy (Nocellara del Belice, Nocarella Messinese, Tonda Iblea, Pennulara, Koroneiki, Biancolilla, Cima di Melfi, Coratina), six came from Portugal/Spain (Picudo, Hojiblanca, Picual, Pajarera, Arbequina, Cuquillo) and four mono-varieties came from Greece (Koroneiki, Biancolilla, Kolovi, Kothreiki).

### 2.3. Principal Component Analysis

**Figure 6:**
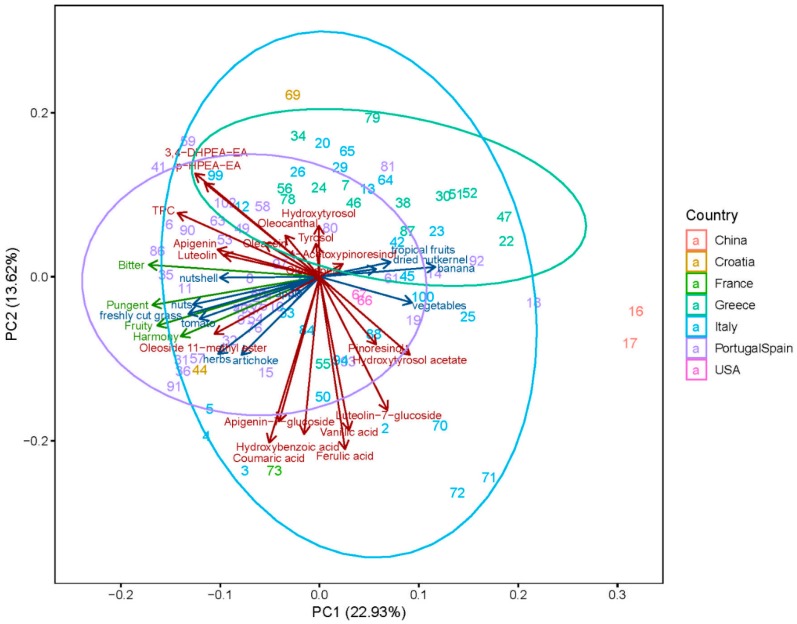
Principal component analysis for all of the analyzed sensory descriptors and the bioactive compounds. All of the twenty targeted ∆ phenolic compounds, the ∆ sensory and the ∆ aroma descriptors are showed.

Principal component analysis (PCA) was used to identify the distribution of the samples, their bioactive compounds and their aroma and sensory descriptions. The first two principal components explained 36.5% of the total variance with PC1 = 22.9% and PC2 = 13.6% (Figure 6). Both sensory and phenolic attributes do not contribute to the overall variance. The chemical and sensory properties of olive oils are too complex to be reduced to two dimensions.

The sensory attributes contribute strongly to the first component, whereas many polyphenols rather contribute to the second component. The second component consisted of phenolic compounds with components loading on the positive scale such as 3,4-DHPEA-EA, *p*-HPEA-EA, TPC, oleocanthal, hydroxytyrosol, apigenin, oleacein, tyrosol, luteolin, 1-acetoxypinoresinol, and oleuropein.

The PCA detected relationship between bioactive compounds, sensory and aroma descriptors, allowing a country specific classification of olive oil sample. Three clusters could be identified based on the country of origin. The first cluster is formed by the Greece olive oils, the second cluster is formed by the Iberian olive oils and the thirds and rather imprecise and is formed by the Italian olive oils (Figure 6). The larger the number of olive oil samples and their corresponding mono-varieties and blends, the more imprecise the cluster gets. Italian olive oil represents the country with the largest number of olive oil blends, which effects the clustering.

In this study, the PCA reveals pattern that are correlating with the sample characteristics, such as olive oil preference. It shows, that especially olive oil located in the right middle area of the plot forms a cluster. These olive oil becomes more preferred and where rated rather highly at the annual competition of the “Olive Oil Award–Zurich” (2017). Hereby, especially olive oil with the following No. have been awarded with the gold medal: 5, 6, 8, 36, 37, 41, and 44. Olive oils awarded with the silver medal were as followed: 3, 9, 11, 35, 40, 60, 62, 76, 77, 86, and 89. Olive oils awarded with the bronze medal were as followed: 4, 12, 15, 31, 32, 43, 57, 73, 91, 98, and 99. As can be seen, the sensory descriptors harmony, intensity, fruity, and pungent are also located in the right middle area of the PCA plot as well as the aroma descriptors freshly cut grass, nuts, tomato, herbs, artichoke, and nutshell. From this, it can be deduced that the stronger the sensory and aromatic attributes of the oil are, the more preferred it will be.

Exploratory PCA studies done by Farrés-Cebrián et al. [34] could differentiate between 100% Arbequina and 100% Picual olive oils based on fourteen simple polyphenols and phenolic acids. Furthermore, they discussed to resolve authentication and adulteration issues carried out by partial last square regression.

Understanding the clustering of phenolic compounds and the sensory discriptors, luteolin, apigenin, and oleoside 11-methylester in particular play a decisive role here. As the only polyphenol, it forms a common cluster with the sensory descriptors harmony, intensity, fruity, and pungent. This behavior indicates that especially this phenolic compound can be used as marker for the mentioned quality parameters. Besides, oleoside 11-methylester showed a good correlation with the aroma descriptor herbs, tomato and artichoke and can also form a separate cluster. In addition, the aroma descriptor tropical fruits was best correlated with the olive oil ripening stage. However, there is not a simple linear relationship between the sensory attributes and the phenolic compounds. In addition, the determination of the TPC, as a common colorimetric assay with a faster analysis time and a lower cost, provides a good correlation according to the olive oil awarded cluster.

## 3. Materials and Methods

### 3.1. Materials and Reagents

Standards of the following phenolic compounds were obtained from PhytoLab GmbH & Co. KG (Vestenbergsgreuth, Germany): apigenin, apigenin-7-*O*-glucoside, trans-cinnamic acid, *p*-coumaric acid, trans-ferulic acid, protocatechuic acid, maslinic acid, vanillic acid, tyrosol, hydroxytyrosol, hydroxybenzoic acid, hydroxytyrosol acetat, oleanolic acid, oleoside-11-methylester, luteolin, luteolin-7-*O*-glucoside, (+)-pinoresinol, 1-acetoxypinoresinol, oleuropein, oleacein, *p*-HPEA-EA, and oleocanthal. Acetonitrile, methanol, water, and formic acid (LC-MS grade) were purchased from Sigma Aldrich Chemie GmbH (Buchs, Switzerland). 2 M Folin-Ciocalteau reagent and anhydrous sodium carbonate (both from Sigma) were used to measure the TPC.

### 3.2. Plant Material

All olive oil samples were obtained from the project “Olive Oil Award – Zurich” (2017), an annual competition, based on the professional sensory evaluation of olive oils by a trained panel of olive oil experts (Swiss Olive Oil Panel, SOP), founded in 2002 by the Zurich University of Applied Sciences (ZHAW), Switzerland. One hundred samples were submitted from eight different countries: Italy (*n* = 28), Spain (*n* = 27), Greece (*n* = 15), Portugal (*n* = 11), Croatia (*n* = 3), France (*n* = 1), USA (*n* = 3), and China (*n* = 2). Seven samples were blends from different European countries and were therefore labeled with the origin ‘European Union’. In general, the olive oils were comprised of mono-varieties (*n* = 62) or blends (*n* = 35). The mono-varieties could be categorized as Picual (*n* = 17), Koroneiki (*n* = 12), Hojiblanca (*n* = 10), Nocellara (*n* = 9), Cobrançosa (*n* = 8), and Arbequina (*n* = 6). However, according to the sensory evaluation by the trained panel of olive oil experts seven out of one hundred olive oils were awarded at the annual competition with a gold medail. Additionally eleven silver medails and eleven bronze medails were awarded, whereby the remaining seventy-one olive oil samples received no particular positiv evaluation.

### 3.3. Sample Preparation

#### Plant Material

A sample of 5 g olive oil was weighed into a 50 mL centrifuge tube and dissolved with 5 mL hexane to reduce the emulsifying effect during the extraction and to improve the separation of the two-phases during the centrifugation. Phenolic compounds were extracted twice with 3 mL methanol:water (60:40, *v*/*v*) and once with acetonitrile:water (80:20, *v*/*v*). After each extraction step, the two phases were separated by centrifuging at 2880× *g* for 5 min (Centrifuge 5810, Vaudaux-Eppendorf AG, Schönenbuch, Switzerland) and the hydroalcoholic phases were combined. After centrifugation, the oils were decanted and filtered using ChromabondTM PTL columns (Macherey-Nagel GmbH & Co. KG, Oensingen, Switzerland). The hydroalcoholic phase was further evaporated to dryness under gaseous nitrogen at 20 °C with a sample concentrator (Portmann Instruments AG, Biel-Benken, Switzerland). For further RP-HPLC-DAD-ESI/MS analysis, samples were subsequently dissolved in 200 µL methanol:water (60:40, *v*/*v*) and 200 µL acetonitrile:water (80:20, *v*/*v*), and held at −20 °C before analysis. Each sample was prepared in triplicate and each one was analyzed three times (mean ± SD).

### 3.4. Quantification of Chemical Components Using LC-DAD/ESI-MS

Identification of chemical components of olive oil extracts was confirmed by RP-LC-DAD-ESI/MS. This was performed on an Agilent 1200 series liquid chromatography and single quadrupole mass spectrometer with electrospray ionization interface (LC-MS 6120, G6100 series, Agilent Technologies AG, Waldbronn, Germany). The olive oil extracts were analyzed using a gradient mixture of water:formic acid (99.9:0.1, *v*:*v*) (solvent A) and acetonitrile:water:formic acid (94.9:5:0.1, *v*:*v*:*v*) (solvent B). A 3.0 × 150 mm Eclipse XDB-C18 (3.5 µm) column (Agilent Technologies AG) was applied. The separation was affected using a linear gradient at 60 °C with a flow of 0.2 mL/min as follows: 10% B at 0–3 min, 10–12% B at 3–8 min, 12–23% B at 8–16 min, 23–25% B at 16–21 min, 25–30% B at 21–26 min, 30% B at 26–30 min, 30–36% B at 30–42 min, 36–70% B at 42–55 min, 70–98% B at 55–60 min, 98% at 60–65 min. The re-equilibration time was 7 min and the injection volume was 6 µL.

For ESI/MS analysis, the positive capillary voltage was set at 4000 V and the negative at 3000 V. The drying gas temperature was 330 °C and the drying gas flow 11 mL/min. The samples were analyzed using a full scan from 100–2000 *m*/*z* in positive ionization mode.

UV-spectra, retention times and characteristic fragmentation patterns were obtained using standard substances for identification of chemical components. LC-DAD analysis was performed by monitoring different wavelengths. Phenylethanoids like tyrosol, hydroxytyrosol, hydroxybenzoic acid, and hydroxytyrosol acetat were detected at 275 nm, flavones like apigenin, apigenin-7-*O*-glucoside, luteolin, and luteolin-7-*O*-glucoside at 360 nm, phenolic acids like *p*-coumaric acid, ferulic acid, vanillic acid at 320 nm, and oleosidic compounds like oleanolic acid, oleoside-11-methylester, oleuropein, oleacein and oleocanthal at 240 nm. Lignans like pinoresinol and 1-acetoxypinoresinol were detected at 210 nm (Table 2).

The purity of the standards was 95% or higher. All standards were prepared as stock solutions at 1 mg/mL in methanol for phenylethanoids, hydroxycinnamates, terpenoids, and DMSO for the flavonoids and their glycosides. The stock solutions were stored at −20 °C and used to prepare working mix solutions by diluting the appropriate volume of the stocks in methanol, in the range of 0.01 mg/L to 0.6 mg/L.

### 3.5. Characterization of Chemical Components Using Folin-Ciocalteu Assay

Several techniques had to be combined for a comprehensive determination of the chemical compounds. The analysis of individual phenolic compounds could be effectively performed using LC-DAD/ESI-MS. The advantage of a fast track method such as the photometric Folin-Ciocalteau assay is a quick analysis, yielding results already in a crude matrix. The TPC of the olive oil extracts was determined using the Folin-Ciocalteau assay according to Singleton and Rossi [35] with some modifications according to Pedan and co-workers [36]. Gallic acid was used as a calibration standard and results were expressed as milligrams of gallic acid equivalent per gram (mg GAE/g).

### 3.6. Statistical Analysis

Data analysis was performed using the statistics software R (Version 3.5.1, R Core Team, 2018) [37]. Data in Figure 3, Figure 4 and Figure 5 were transformed and scaled for better visibility. Using the R package BestNormalize, the Box-Cox transformation was applied to achieve data that is closer to a standard normal distribution with mean 0 and standard deviation 1. The analysis of variance (ANOVA) and Tukey’s Honestly Significant Difference (HSD) for pairwise post-hoc tests were used to evaluate differences between the fruitiness intensity light, light/medium, medium, and medium/intense and the TPC as well as some individual phenolic compounds (Figure 3, Figure 4 and Figure 5). The normality assumption of the residuals was tested in all these cases with the Shapiro-Wilk test and the assumption of homoscedasticity with the Bartlett’s test. For the analysis of individual phenolic compounds like oleoside-11-methyl ester with different intensities, the Kruskal-Wallis test and the pairwise Wilcoxon test for post-hoc tests were applied as the normality assumption of the residuals was violated in that case (Figure 3). To evaluate neutral groupings in the evaluated data, principal component analysis (PCA) was carried out (Figure 6). Furthermore, correlations have been made with the sensory description in combination with the individual phenolic compounds (Supplementary Material).

## 4. Conclusions

Within the present study, a tool has been created to segment, a wide and heterogeneous trade category by the means of their phenolic composition and their sensory properties. In addition, the created knowledge of individual phenolic compounds to health, flavor and taste, gains valuable information for costumers and can finally help to promote the health claim concerning EVOO.

Also Roselli et al. [38] postulated in their scientific commentary a benefit for customers concerning the phenolic content as a reduced consumers’ information about the product, which can create value in the olive oil growing sector.

The present study investigated the phenolic composition and the sensory profile of EVOO from different varieties. It has been showed, that all of the detected EVOOs were too low to provide the minimum intake of 5 mg hydroxytyrosol per serving olive oil, which is required to have an antioxidant effect in a balanced diet. Besides, four out of hundred EVOOs could provide a TPC higher than 250 mg/kg.

Individual phenolic compounds can be used to differentiate between cultivars, ripening stages, fruitiness intensities, and sensory characteristics. Especially oleoside 11-methylester could be identified as indicator for correlations for the aroma descriptors freshly cut grass, leaves, and nuts.

However, with a large-scale sensory panel, sensory attributes are much more difficult to collect than the determination of phenolic compounds, where the sample preparation has keeps quick and easy, individual phenolic compounds can be used as indicators for sensory description. The results could serve as a reference to both traders and consumers when purchasing olive oils for consumption.

The original mission to generate a predictive model to understand the influence of sensory descriptors to TPC and individual phenolic can be done by an exploratory PCA. Here, data obtained by HPLC-DAD demonstrated that this method can be used for olive oil discrimination in terms of ripening stage, fruitiness intensity, and especially in terms of consumer acceptance and preference.

A fingerprint identification method has been developed for the biochemical compounds of olive oil based on its LC/MS profile. Acceptable sensitivity (limit of detection (LOD) values down to 30 mg/L and limit of quantification (LOQ) values down to 87 mg/L), linearity (R^2^ higher than 0.99) and good run-to-run and day-to-day precisions were obtained.

## Figures and Tables

**Table 1:** Statistical distribution of all analyzed biochemical compounds found in olive oil. Data are presented as mean ± SD (*n* = 3).

Phenolic Compounds [mg/kg]	Min	Q1	Median	Q3	Max
TPC	52.2	135.2	166.7	207.7	315.2
Hydroxytyrosol	0.6	4.9	7.2	11.4	53.7
Hydroxybenzoic acid	0.02	0.04	0.05	0.07	0.2
Tyrosol	0.2	0.6	0.8	1.2	6.6
Vanillic acid	0.2	0.4	0.49	0.7	4.4
Oleacein	1.5	48.5	83.6	118.1	239.9
Oleocanthal	0.55	3.72	4.74	6.52	35.3
Oleuropein	1.7	12.1	21.9	34.40	82.5
*p*-HPEA-EA	0.1	1.2	1.8	2.8	8.1
Hydroxytyrosol acetate	0.00	0.03	0.07	0.19	2.09
Oleoside 11-methyl ester	0.9	8.4	11.9	20.2	92.2
Oleuropein	1.18	3.88	7.05	10.18	37.8
Oleocanthal	11.8	97.4	155.8	208.9	358.2
Coumaric acid	0.04	0.15	0.29	0.53	2.54
Ferulic acid	0.00	0.01	0.02	0.04	0.48
Luteolin-7-*O*-glucoside	0.01	0.02	0.02	0.03	0.18
Apigenin-7-*O*-glucoside	0.01	0.02	0.02	0.03	0.15
Luteolin	0.5	1.6	2.5	3.7	9.4
Apigenin	0.1	0.7	1.0	1.5	8.2
Pinoresinol	1.1	3.1	4.2	5.6	11.9
1-Acetoxypinoresinol	4.7	21.6	25.8	31.7	90.6

**Table 2:** Mass spectrometric identification results.

No.	Identification	RT [min]	Calibration Curve	UV [nm]	LOD [mg/L]	LOQ [mg/L]	Formula	MW [g/mol]	[M + H]^+^ [*m*/*z*]	Major Fragments [M + H]^+^	
1	(+)-Pinoresinol	36.2	y = 24767x + 53.46	210	7.0	21.3	C_20_H_22_O_6_	358.38	359.15	341.0	175.0
2	Oleoside 11-methyl ester	18.7	y = 5698.6x + 39.59	240	14.9	45.1	C_17_H_24_O_11_	404.37	405.14	165.1	151.1
3	Oleuropein	30.1	y = 4259.4x − 21.44	240	9.6	29.0	C_25_H_32_O_13_	540.51	541.19	137.1	360.9
4	Oleocanthal	39.6	y = 4422.9x + 22.99	240	13.2	40.0	C_17_H_20_O_5_	304.34	305.14	345.1	121.1
5	Hydroxytyrosol	10.2	y = 3434.8x + 0.23	275	2.3	6.9	C_8_H_10_O_3_	154.17	155.07	137.1	119.1
6	Hydroxybenzoic acid	12.9	y = 12683x − 6.11	275	15.4	46.6	C_7_H_6_O_3_	138.12	139.04	-	-
7	Tyrosol	14.0	y = 31878x − 71.75	275	8.1	24.5	C_8_H_10_O_2_	138.16	139.07	121.1	103.1
8	Vanillic acid	15.5	y = 2266.5x − 13.72	275	28.7	87.0	C_8_H_8_O_4_	168.15	169.05	125.1	110.0
9	Hydroxytyrosol acetate	25.3	y = 25238x + 97.11	275	4.7	14.2	C_10_H_12_O_4_	196.20	197.08	137.1	119.1
10	*p*-Coumaric acid	20.7	y = 19893x + 84.60	320	10.9	32.9	C_9_H_8_O_3_	164.16	165.05	147.0	119.1
11	trans-Ferulic acid	21.6	y = 16107x + 69.68	320	10.3	31.4	C_10_H_10_O_4_	194.18	195.06	177.0	195.1
12	Luteolin-7-*O*-glucoside	21.8	y = 12204x + 24.43	360	7.4	22.4	C_21_H_20_O_11_	448.38	449.11	449.0	-
13	Apigenin-7-*O*-glucoside	24.5	y = 8639x + 29.64	360	8.8	26.5	C_21_H_20_O_10_	432.38	433.11	433.1	271.1
14	Luteolin	31.5	y = 17000x + 49.81	360	8.0	24.2	C_15_H_10_O_6_	286.24	287.06	287.0	-
15	Apigenin	37.4	y = 15837x + 134.01	360	16.6	50.3	C_15_H_10_O_5_	270.24	271.06	271.0	-

## Supplementary Materials

Supplementary materials are available online.
