# Supplementary figures and images for: Characterization of Phenolic Compounds and Their Contribution to Sensory Properties of Olive Oil
Source: Molecules. 2019 May 28;24(11):2041. doi: 10.3390/molecules24112041 (PMC6600435; doi:10.3390/molecules24112041)

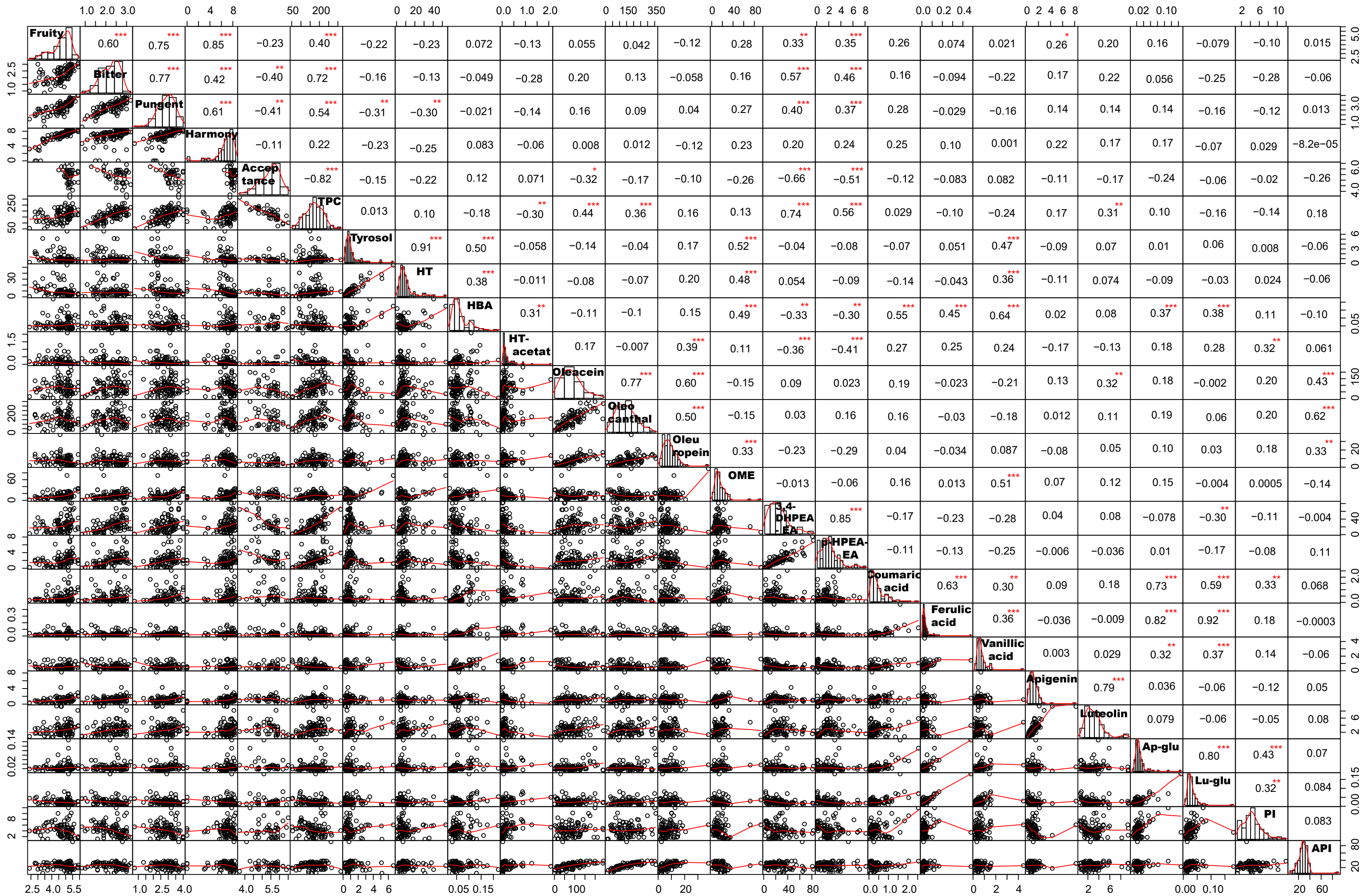

Supplement: Supplementary file 1 [file molecules-24-02041-s001.pdf]
